# Effect of Diquat on gut health: molecular mechanisms, toxic effects, and protective strategies

**DOI:** 10.3389/fphar.2025.1562182

**Published:** 2025-05-12

**Authors:** Cheng He, Guorong Cai, Yingmao Jia, Rong Jiang, Xiaolan Wei, Ning Tao

**Affiliations:** Department of Emergency, Suining Central Hospital in Sichuan Province, Suining, Sichuan, China

**Keywords:** Diquat, gut health, molecular mechanisms, toxic effects, protective strategies

## Abstract

Diquat is a widely used bipyridyl herbicide that is extensively applied in agricultural production and water management due to its high efficacy in weed control. However, its environmental persistence and the toxic effects it induces have raised widespread concern. Studies show that Diquat primarily enters the body through the digestive tract, leading to poisoning. The core mechanism of its toxicity involves reactive oxygen species (ROS)-induced oxidative stress, which not only directly damages the intestinal barrier function but also exacerbates inflammation and systemic toxicity by disrupting the balance of the gut microbiota and the normal production of metabolic products. This review systematically summarizes the physicochemical properties of Diquat, with a focus on analyzing the mechanisms by which it damages the gut tissue structure, barrier function, and microbiota after digestive tract exposure. The aim is to provide theoretical support for a deeper understanding of Diquat’s toxic mechanisms and its digestive tract-centered toxic characteristics, laying a scientific foundation for the development of effective interventions and protective strategies against its toxicity.

## 1 Introduction

Diquat (1,1′-ethylene-2,2′-bipyridyl) is a bipyridyl herbicide widely used in agriculture and non-cultivated land weed control due to its rapid action and broad-spectrum efficacy (Wilkinson, 1969). Its high water solubility and stability in acidic and neutral environments contribute to its persistent environmental residues, although it can degrade into simple organic compounds and inorganic salts under alkaline conditions (Jones and Vale, 2000). Although Diquat exhibits lower acute toxicity compared to the related herbicide paraquat, it still poses a significant threat to ecosystems due to its potential for bioaccumulation and long-term persistence in soil and water bodies (Wilkinson, 1969; Akhavein and Linscott, 1968).

The toxicity of Diquat arises from its redox cycling capability, which generates reactive oxygen species (ROS) such as superoxide anions (O_2_^−^·) and hydroxyl radicals (OH·) (Ren et al., 2024; Fussell et al., 2011; Aloise et al., 2022). These ROS cause oxidative damage to lipids, proteins, and DNA, ultimately leading to multi-organ dysfunction. The kidney is the primary target organ, where Diquat can induce acute tubular necrosis, mitochondrial dysfunction, and apoptosis (Xing et al., 2020; Yu et al., 2022; Chen et al., 2024; Lai et al., 2024; Wu et al., 2022a). In addition, lung and nervous system injuries have also been reported, which in severe cases can progress to multiple organ failure (Ren et al., 2024; Sun et al., 2020; Zhang et al., 2022). However, existing studies have largely focused on systemic toxicity following absorption, overlooking the gastrointestinal tract as both an exposure route and a direct target organ.

The intestine is a critical interface for nutrient absorption, immune regulation, and microbial symbiosis (Takiishi et al., 2017). The integrity of the intestinal epithelial barrier is maintained by tight junction proteins such as ZO-1 and occludin, which prevent pathogen invasion and systemic inflammation (Gieryńska et al., 2022). The gut microbiota further modulates host metabolism, immune responses, and resistance to oxidative stress (Ma et al., 2025). Disruption of barrier function, dysbiosis, or chronic inflammation can destabilize this balance and is closely associated with systemic diseases such as metabolic disorders and neurodegenerative conditions (Hrncir, 2022; Zhao et al., 2023). Therefore, intestinal health serves as a central line of defense against damage from exogenous toxicants.

Recent studies have identified the gut as a key target of Diquat toxicity (Cao et al., 2020; Clark and Hurst, 1970; Rawlings et al., 1994). Oral ingestion, the primary route of exposure, allows Diquat to directly damage intestinal epithelial cells, disrupt tight junctions, and trigger ROS-mediated inflammatory responses (Huang et al., 2025; Jin et al., 2021; Xu et al., 2023). Diquat also alters gut microbiota composition, reducing the abundance of *Lactobacillus* while enriching pathogenic bacteria, thereby exacerbating both intestinal and systemic toxicity (Fu et al., 2021; Wen et al., 2024a). Moreover, Diquat promotes a vicious cycle of oxidative stress and immune dysregulation by inhibiting antioxidant defenses and activating pro-inflammatory pathways such as NF-κB and MAPK (Xu et al., 2023; Xu Q. et al., 2022). Nevertheless, the molecular mechanisms linking gut-specific injury to systemic organ failure remain unclear.

Current research on Diquat toxicity has been overly focused on liver and kidney dysfunction, while the role of intestinal mechanisms in systemic pathology remains insufficiently integrated. Key unresolved questions include: (1) How does Diquat-induced disruption of the intestinal barrier facilitate toxin translocation and injury to distal organs? (2) Do gut microbiota-derived metabolites modulate Diquat toxicity? (3) Can targeting intestinal integrity or microbial balance alleviate multiple organ dysfunction? Moreover, although antioxidants (e.g., resveratrol) and probiotics have shown promise in preclinical models, the mechanisms by which they restore gut-immune interactions remain to be fully elucidated. This review synthesizes existing evidence on Diquat-induced structural damage to the gut, microbial dysbiosis, and immune activation, highlighting how these processes amplify systemic toxicity. We propose a gut-centered intervention strategy to bridge the knowledge gap between local intestinal injury and systemic effects, offering new perspectives for detoxification and clinical management.

## 2 Physicochemical properties and metabolic process of diquat

### 2.1 Basic chemical structure and properties

Diquat has the full chemical name of 1,1′-ethylene-2,2′-bipyridinium. It is a bipyridyl compound with a molecular formula of C12H12N2 (ionic form) or C12H12Br2N2 (dibromide form), with molecular weights of 184.24 and 344.05, respectively. It exists as odorless yellow crystals, while the commercial products are often dark green or reddish-brown, exhibiting high water solubility (solubility 712 g/L, pH 5.2). Under acidic and neutral conditions, Diquat demonstrates high stability, but it is prone to hydrolysis in alkaline solutions. Its half-life in aquatic environments ranges from 2 to 10 days, primarily degrading into simple organic molecules and inorganic salts (Wilkinson, 1969; Kim et al., 2024; Summers and Black, 1968). These properties confer strong environmental persistence and potential ecological toxicity to Diquat.

### 2.2 Forms of diquat in the environment and its metabolism

In the environment, the form of Diquat primarily depends on the medium in which it is found. In soil, Diquat is easily adsorbed onto soil particles, resulting in low mobility and slow degradation, primarily relying on microbial and chemical processes for breakdown (Kim et al., 2024). In water, Diquat can be removed through photodegradation and microbial activity after binding with suspended particles. Although its degradation rate is relatively fast, the degradation products may pose secondary toxicity to aquatic organisms. Due to Diquat’s accumulation behavior, it may pose long-term threats to organisms and ecosystems in the environment (Summers and Black, 1968).

### 2.3 Absorption, distribution, metabolism, excretion, and organ damage in animals

The primary route of absorption of Diquat is through the digestive tract, with a low absorption rate (<10%) (Duan et al., 2024). Once inside the body, Diquat enters tissues via diffusion or active transport through ion pumps and rapidly distributes throughout the system (Magalhães et al., 2018). Autopsy findings from patients who died from DQ poisoning have shown that tissue (and fluid) concentrations of DQ vary depending on the time of death. For instance, in a patient who died 14 h after ingestion, the concentration of DQ from highest to lowest was: urine > vitreous humor > lungs > liver > brain tissue > kidneys (McCarthy and Speth, 1983). During metabolism, Diquat undergoes a redox cycle to generate highly reactive DQ^+^, which further binds with oxygen molecules to form superoxide anion (O_2_^−^) and other ROSs. These ROSs significantly induce cellular damage, primarily manifested as lipid peroxidation, protein oxidation, and DNA damage, which in turn triggers oxidative stress, leading to disruption of cell membrane structures, mitochondrial dysfunction, and cell apoptosis (Fussell et al., 2011; Awad et al., 1994; Wolfgang et al., 1991; Zhang et al., 2006; Blakeman et al., 1998; Wen et al., 2024b; Gupta et al., 2000). The excretion of Diquat primarily occurs through urine and feces, with most of it being excreted in its unmetabolized parent form (Jones and Vale, 2000; Basilicata et al., 2022; Guo et al., 2023; Magalhães et al., 2018). However, Diquat that is not effectively eliminated continues to induce toxic reactions in the body. The main renal toxicity of Diquat is acute kidney injury (AKI), with ROS being a key pathological inducer in its molecular mechanism. ROS attacks the polyunsaturated fatty acids in the renal tubular cell membranes, leading to lipid peroxidation and membrane structural damage (Chen et al., 2024; Wu et al., 2024). Studies have also found that Diquat induces gasdermin E (GSDME) cleavage through mitochondrial dysfunction, triggering pyroptosis and leading to damage of renal tubular cells (Chen et al., 2024). Additionally, Diquat activates the HMGB1/IκBα/NF-κB signaling axis, inducing the massive release of pro-inflammatory cytokines, which exacerbates renal interstitial inflammation and fibrosis (Huang et al., 2023). Diquat that is not eliminated causes irreversible structural and functional damage to the kidneys through these mechanisms (Yu et al., 2022). In the liver, the toxicity of Diquat is dominated by mitochondrial dysfunction and signaling pathway disorders. Diquat downregulates the expression of SIRT1, and inhibits the activity of mitochondrial complexes (I, II, III, and V), leading to a significant reduction in ATP synthesis, which further induces energy crisis and apoptosis in hepatocytes (Jia et al., 2020; Zhang et al., 2020). At the same time, Diquat promotes hepatocyte apoptosis by inhibiting the expression of anti-apoptotic proteins and enhancing the activity of pro-apoptotic factors (Fu et al., 2001; Hong et al., 2009; Luo et al., 2024). Moreover, Diquat increases the expression of autophagy-related proteins (such as Beclin-1 and LC3-II), but the abnormal accumulation of p62 indicates that autophagy function is not effectively completed, further aggravating the stress response and damage in hepatocytes (Chen et al., 2021). Diquat-induced ROSs also stimulate hepatocytes to release pro-inflammatory cytokines (such as IL-1β and IL-18), enhancing liver inflammation and promoting liver damage (Luo et al., 2024). Although the respiratory system is not the primary target of Diquat, its toxicity still causes damage through ROS and inflammatory signaling pathways. Diquat significantly activates the Nrf2 signaling axis in lung tissue, which, on the one hand, induces antioxidant defense, but on the other hand, exacerbates oxidative stress and damages lung tissue due to the excessive generation of ROS (Fussell et al., 2011; Sun et al., 2020; Wu et al., 2012). Diquat induces systemic oxidative stress and inflammatory responses throughout the body, leading to multiple organ dysfunction syndrome (MODS) (Huang et al., 2021; Yoshioka et al., 1992).

## 3 The relationship between diquat and gut dysfunction

**FIGURE 1:**
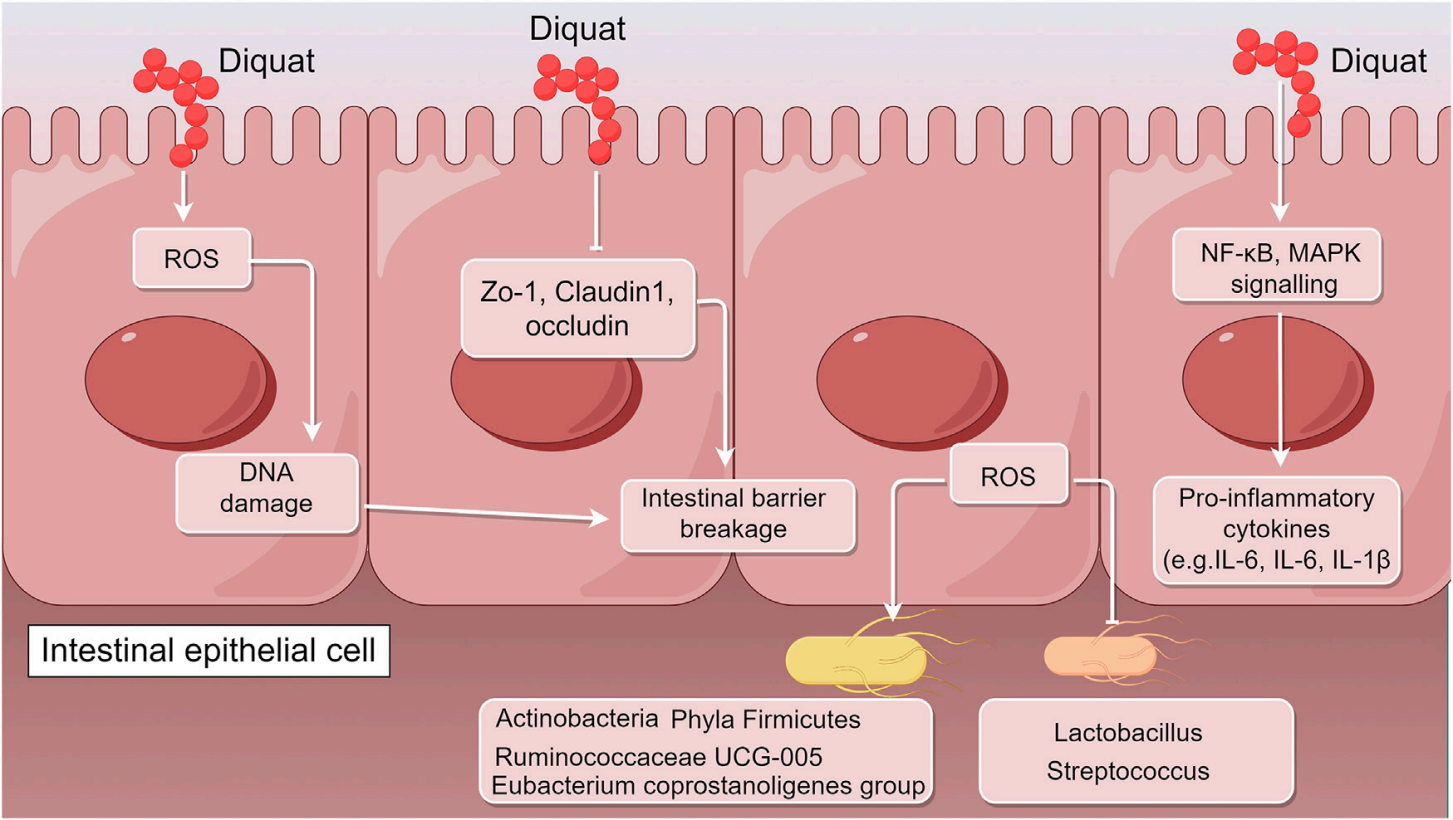
Schematic illustration of the molecular mechanisms underlying Diquat-induced intestinal dysfunction (by Figdraw). After entering the body primarily through the digestive tract, Diquat first acts on intestinal tissues, triggering a range of pathological responses including oxidative stress, barrier disruption, microbial dysbiosis, and immune imbalance. Its toxic effects are manifested through several pathways: Diquat induces the generation of ROS, significantly downregulates the expression of tight junction proteins (ZO-1, occludin, claudin-1), and increases intestinal permeability. ROS also activate inflammatory pathways such as NF-κB and MAPK, leading to the excessive release of pro-inflammatory cytokines (TNF-α, IL-6, IL-1β), thereby forming a vicious cycle of inflammation and barrier damage. Additionally, beneficial bacteria (e.g., *Lactobacillus*) are reduced, while pathogenic bacteria increase in abundance.

The toxic mechanism of Diquat focuses on multi-organ damage induced by ROS (Ren et al., 2024; Chen et al., 2024; Jin et al., 2021; Luo et al., 2024; Qu et al., 2024). However, it is worth noting that existing literature reports indicate that the main route of Diquat poisoning is through ingestion, while other routes of exposure are relatively rare. Therefore, the gut, as the main exposure target for Diquat, plays a crucial role in the poisoning process. Diquat not only directly damages the tissue structure of the gut but also exacerbates systemic toxicity by disrupting the gut microbiota and impairing gut immune homeostasis. The specific mechanisms of these effects include oxidative stress, abnormal activation of inflammatory signaling pathways, autophagy dysregulation, and barrier function disruption (Cao et al., 2020; Xu et al., 2023; Xu Q. et al., 2022; Wen et al., 2024b; Cao et al., 2018; Feng et al., 2023; Hao et al., 2021; Liang et al., 2023; Wu et al., 2019). The following will focus on the latest research progress regarding the impact of Diquat on gut tissue structure, microbiota, and immune homeostasis, as well as its potential molecular mechanisms, providing theoretical support for further exploration of its toxicity mechanisms and intervention strategies (Figure 1).

### 3.1 Specific effects of diquat on gut tissue structure

The damage to the intestinal epithelial barrier caused by Diquat is one of its core toxic effects, primarily manifested as the disruption of gut barrier function and the loss of mechanical barrier integrity. At the molecular level, Diquat significantly downregulates the expression of tight junction proteins (such as ZO-1, claudin-1, and occludin) by inducing oxidative stress. These tight junction proteins are key components in maintaining the barrier function between intestinal epithelial cells. A reduction in their expression leads to the loosening of cell-cell connections, significantly increasing intestinal permeability. This damage to the mechanical barrier allows toxins and pathogens in the intestinal lumen to more easily penetrate the barrier and invade tissues, further exacerbating intestinal inflammation (Jin et al., 2021; Cao et al., 2018; Liang et al., 2023; Cao et al., 2019; Wen et al., 2020). Additionally, Diquat activates the NF-κB signaling pathway, leading to the massive release of pro-inflammatory cytokines (such as TNF-α, IL-6, and IL-1β). These pro-inflammatory cytokines not only trigger inflammatory responses but also further suppress the expression of tight junction proteins, thereby creating a vicious cycle of inflammation and barrier damage (Xu Q. et al., 2022; Xu et al., 2023).

Oxidative stress is another key mechanism underlying the toxic effects of Diquat (Cao et al., 2020; Magalhães et al., 2018; Cao et al., 2018; Feng et al., 2023; Liang et al., 2023). The molecular action of Diquat generates a large amount of ROS through its enzymatic reactions, including superoxide anion (O_2_^−^), hydrogen peroxide (H2O2), and hydroxyl radicals (OH·). These ROSs are powerful oxidants that can directly cause oxidative damage to the cell membranes, proteins, and DNA of intestinal cells (Afzal et al., 2023). Diquat significantly increases the levels of malondialdehyde (MDA) in intestinal tissues, a marker of lipid peroxidation, while inhibiting the activity of antioxidant enzymes such as superoxide dismutase (SOD) and glutathione peroxidase (GSH-Px), further weakening the cell’s defense against oxidative stress (Zhang et al., 2021). Moreover, excess ROSs also promote the expression of pro-inflammatory factors by activating the NF-κB pathway, enhancing the inflammatory response, and thereby exacerbating intestinal damage (Zhang et al., 2021).

In terms of mitochondrial function, Diquat induces excessive ROS production, which directly damages mitochondrial structure and function (Choi et al., 2018; Gou et al., 2024; Nisar et al., 2015). A significant decrease in mitochondrial membrane potential (MMP) is an early sign of mitochondrial dysfunction, indicating impaired mitochondrial oxidative phosphorylation and energy metabolism (Khan et al., 2022; Gottlieb et al., 2003). Diquat also induces pathological changes such as mitochondrial swelling, cristae rupture, and vacuolization, further affecting mitochondrial metabolic function. To cope with this damage, cells typically activate mitochondrial autophagy through the PINK1/Parkin signaling pathway, marking and degrading damaged mitochondria to maintain mitochondrial homeostasis. However, excessive autophagy induced by Diquat may lead to further deterioration of mitochondrial function (Cao et al., 2020). Furthermore, Diquat interferes with the function of the endoplasmic reticulum-mitochondria-associated membranes (MAM), disrupting the transport of Ca^2+^ between the endoplasmic reticulum and mitochondria, further weakening mitochondrial activity and intestinal cell metabolic function (Li et al., 2022).

It is worth emphasizing that mitochondria, as key organelles regulating cellular metabolism, signal transduction, and cell fate, play an indispensable role in maintaining the integrity of the intestinal epithelial barrier (Rath and Haller, 2022). Recent studies have shown that mitochondria in intestinal epithelial cells not only contribute to energy supply—particularly through oxidative phosphorylation to produce ATP—but also directly participate in the structural and functional regulation of the mucosal barrier (Wang et al., 2023; Ho and Theiss, 2022). Mitochondrial dysfunction can impair intestinal structure through multiple mechanisms. Firstly, impaired oxidative phosphorylation leads to reduced ATP production, which in turn suppresses the expression and stability of tight junction proteins, disrupts intercellular connections in the intestinal epithelium, and increases intestinal permeability (Song et al., 2023). Secondly, damaged mitochondria release large amounts of ROS and mitochondrial DNA (mtDNA), which act as danger-associated molecular patterns (DAMPs) that activate the NLRP3 inflammasome and NF-κB pathway, amplifying the inflammatory response (Zong et al., 2024). Thirdly, disruption of local oxygen homeostasis compromises the anaerobic environment, resulting in the depletion of beneficial anaerobic bacteria and further weakening the mucosal barrier’s anti-inflammatory and reparative functions (Yan et al., 2023). More critically, in diseases such as inflammatory bowel disease and colorectal cancer, mitochondrial dysfunction is not only a consequence of tissue damage but may also serve as an initiating factor in pathogenesis. By regulating apoptosis, autophagy, and cell phenotype transformation, it contributes to disease progression (Kłos and Dabravolski, 2021).

In terms of intestinal morphology, the toxic effects of Diquat manifest as a significant reduction in villus height and an increase in crypt depth (Chen et al., 2023). These morphological changes directly impair the absorption function of the intestine, affecting the effective absorption of nutrients (Groschwitz and Hogan, 2009; Salvo Romero et al., 2015; Turner, 2009). In summary, Diquat induces mitochondrial dysfunction, leading to marked damage to the intestinal epithelial barrier and tissue structure through multiple pathways, including disrupted energy metabolism, amplified inflammation, and microbial dysbiosis. These findings provide theoretical support for the intestinal toxicity mechanisms of oxidative stress-inducing toxicants.

### 3.2 Specific impact of diquat on the gut microbiota

Diquat’s impact on the gut microbiota manifests as significant dysbiosis, primarily characterized by a reduction in beneficial bacteria and an increase in harmful bacteria. Studies have shown that Diquat markedly reduces the abundance of *Lactobacillus* and *Streptococcus*, both of which, despite potentially playing different roles in regulating intestinal barrier function and immune homeostasis, are important components of a healthy gut microbiota (Wen et al., 2024c; Wu et al., 2020; Wu et al., 2022b; Dominguez et al., 2024; Wang et al., 2022). Meanwhile, Diquat exposure leads to an increased relative abundance of members from the Phylum Firmicutes and Actinobacteria, Ruminococcaceae UCG-005 and Eubacterium coprostanoligenes (Wen et al., 2024a). This imbalance weakens the protective role of beneficial bacteria, while harmful bacteria further promote inflammation and barrier damage (Fu et al., 2021). At the molecular level, Diquat exacerbates dysbiosis through oxidative stress mechanisms. Elevated ROS levels directly inhibit the survival of *Lactobacillus*, while Actinobacteria and Ruminococcaceae exhibit stronger tolerance to the oxidative stress environment, allowing them to proliferate (Fu et al., 2021). In addition, the expression of tight junction proteins (such as occludin, claudin-1, and ZO-1) is significantly reduced, further compromising the intestinal barrier and creating conditions favorable for the overgrowth of harmful bacteria (Chelakkot et al., 2018; He et al., 2023; Ulluwishewa et al., 2011). This dysbiosis also affects the production of intestinal metabolites, such as a significant decrease in indole-3-methanol and butyrate levels, which not only weakens the intestinal anti-inflammatory and antioxidant capabilities but also impacts the repair functions of the intestinal barrier (Fu et al., 2021). Through the disruption of the microbiota-metabolite axis, Diquat not only alters the microbiota structure but also disturbs gut signaling and metabolic regulation by influencing the production of metabolites (such as uridine and indole) (Fu et al., 2021; Saito, 1986). The reduction of *Lactobacillus* and associated metabolites is directly related, and the absence of these metabolic products further impairs the microbiota’s antioxidant and anti-inflammatory abilities (Fu et al., 2021). Overall, Diquat shapes an ecological environment favorable to harmful bacteria through oxidative stress and the disruption of the microbiota-metabolite axis, thereby exacerbating the dysbiosis of the gut microbiota.

### 3.3 Specific effects of diquat on gut immune homeostasis

Diquat significantly increases the secretion of pro-inflammatory cytokines (such as TNF-α, IL-6, and IL-1β), exacerbating chronic inflammatory responses in the gut (Xu Q. et al., 2022; Xu et al., 2023; Wen et al., 2024c; Wang et al., 2019; Nong et al., 2023). Oxidative stress not only directly activates intestinal macrophages but also further amplifies the intensity of the pro-inflammatory response (Zhang et al., 2021). Molecular mechanism studies show that Diquat-induced ROSs promote the phosphorylation levels of IκB, NF-κB, and ERK1/2, thereby upregulating the gene expression of pro-inflammatory cytokines (Xu et al., 2023). In addition, Diquat enhances the phosphorylation levels of key molecules in the MAPK signaling pathway, including p38, ERK1/2, and JNK, further amplifying the cascade effect of inflammation. These mechanisms collectively drive the persistent inflammatory state in the gut (Wu et al., 2019; Xu Q. et al., 2022; Xu et al., 2023; Liu et al., 2023). Notably, anti-inflammatory therapy studies have shown that antioxidants such as vitamin D3 and resveratrol can significantly alleviate Diquat-induced intestinal inflammation, offering potential therapeutic strategies for intervention (Zhang et al., 2021; Xun et al., 2021; Prakash et al., 2024).

In addition, Diquat significantly weakens the immune barrier function of the intestinal mucosa (Cao et al., 2018; Cao et al., 2019; Gou et al., 2024; Chen et al., 2023; Xun et al., 2021; Chen et al., 2022). This is specifically manifested by reduced secretion of immunoglobulins (such as IgA and IgG) and the downregulation of tight junction proteins in the intestine (such as ZO-1, claudin-1, and occludin), leading to increased intestinal permeability and impaired mucosal barrier function (Wen et al., 2020; Wang et al., 2021). This indicates that Diquat-induced oxidative stress not only triggers chronic inflammation but also causes multiple damages to the intestinal barrier through immune regulatory dysfunction, exacerbating the systemic risks to gut health.

## 4 The mechanisms of antioxidants and probiotics in protecting the intestinal barrier

Resveratrol has shown significant effects in antioxidant activity and intestinal barrier protection by activating the Nrf2 signaling pathway. Its mechanisms of action include enhancing the activity of antioxidant enzymes (such as SOD, GSH-Px, and HO-1), thereby significantly reducing the levels of ROS induced by oxidative stress (Xun et al., 2021; Zhou et al., 2022). Additionally, resveratrol regulates the AhR/Nrf2 pathway, inhibiting the expression of pro-inflammatory factors (such as TNF-α and IL-6), while increasing the abundance of tight junction proteins (such as occludin, claudin-1, and ZO-1), thereby repairing the damaged intestinal barrier function (Xun et al., 2021; Zhou et al., 2022). Further studies have shown that resveratrol also induces mitophagy, alleviating mitochondrial damage and providing multiple layers of protection to mitigate inflammation and support gut health (Cao et al., 2019).

Taurine plays a crucial role in protecting the intestinal barrier, primarily by significantly enhancing the expression of tight junction proteins (such as claudin-1, ZO-1, and occludin), thereby improving intestinal barrier function (Wen et al., 2020). Additionally, taurine effectively alleviates oxidative stress-induced damage to the intestines by regulating immune responses, demonstrating its unique advantage in maintaining intestinal homeostasis (Wen et al., 2020).

Stevioside, a natural antioxidant, protects the intestinal barrier by inhibiting the NF-κB and MAPK signaling pathways. Studies have shown that stevioside significantly increases the activity of antioxidant enzymes (such as T-SOD, CAT, and GSH-Px) and downregulates the expression of pro-inflammatory factors (such as IL-6 and TNF-α), thereby reducing inflammatory responses (Xu et al., 2023). Furthermore, stevioside maintains the integrity of the intestinal barrier by enhancing the expression of tight junction proteins and reducing the permeability of intestinal cells (Xu et al., 2023).

*Lactobacillus* and their extracellular vesicles (EVs) have demonstrated significant antioxidant effects and gut barrier protection by regulating the Nrf2 signaling pathway (Feng et al., 2023; Hao et al., 2021; Qiao et al., 2020; Wang et al., 2013; Xu et al., 2018). For example, Pediococcus pentosaceus ZJUAF-4 and other *Lactobacillus* can improve the composition of gut microbiota while activating Nrf2, thereby significantly reducing ROS produced by oxidative stress (Hao et al., 2021). Additionally, these probiotics and their EVs can maintain the expression of tight junction proteins, effectively repairing gut barrier function and reducing oxidative stress-induced gut damage (Feng et al., 2023). These findings provide new strategies and approaches for the prevention and treatment of gut-related diseases.

The above studies suggest that different types of antioxidants and probiotics, through regulating oxidative stress, inflammatory responses, and the expression of tight junction proteins, work together to protect and repair the gut barrier. This provides scientific evidence for improving gut health and treating related diseases.

## 5 Discussion

As a bipyridyl herbicide, Diquat has traditionally been studied for its toxic mechanisms primarily in relation to oxidative stress-induced damage in organs such as the liver and kidneys. However, recent studies have increasingly revealed that the intestine is not only the primary route of exposure but also a central target of its toxic effects (Cao et al., 2020; Jin et al., 2021; Liang et al., 2023; Xu X. et al., 2022). This review summarizes the mechanisms by which Diquat disrupts intestinal structure, microbial balance, and immune homeostasis, and explores how these local injuries may amplify systemic toxicity through the “gut–organ axis”. Notably, although Diquat has been banned in regions such as the European Union and the United Kingdom due to its ecological toxicity and health risks (EC Regulation No 1107/2009), it is still used for weed control in agriculture and non-cultivated land in certain developing countries. Moreover, residual Diquat in the environment may continue to pose long-term threats to ecosystems and human health via the food chain. Therefore, in-depth investigation of its toxicological mechanisms remains essential for ecological restoration in contaminated areas, clinical management of poisoning cases, and toxicity assessment of structurally related herbicides.

Following oral intake, Diquat directly targets intestinal epithelial cells, where it generates excessive ROS through redox cycling. This leads to the downregulation of tight junction proteins such as ZO-1, occludin, and claudin-1, thereby significantly increasing intestinal permeability (Jin et al., 2021; Cao et al., 2019). The resulting barrier dysfunction facilitates the translocation of endotoxins and unmetabolized Diquat molecules from the gut lumen into the systemic circulation, subsequently triggering a systemic inflammatory response via activation of the TLR4/NF-κB signaling pathway (Cui et al., 2023; Huang et al., 2024; Liu et al., 2021; Zhan et al., 2025). In this process, the gut microbiota plays a pivotal “amplifier” role: Diquat inhibits the proliferation of beneficial bacteria such as *Lactobacillus*, while promoting the enrichment of potentially pathogenic taxa such as Actinobacteria and Ruminococcaceae, leading to a reduction in key microbial metabolites including indole-3-methanol, 5-hydroxyindole-3-acetic acid, and uridine (Fu et al., 2021). These small molecules are normally involved in maintaining intestinal barrier integrity and immune tolerance through activation of the aryl hydrocarbon receptor (AhR). Their depletion exacerbates mucosal damage and inflammatory cascades (Kahalehili et al., 2020; Dang et al., 2023). This resulting cycle of “oxidative stress–microbial dysbiosis–inflammatory amplification” may serve as a key driving force in the development of MODS.

Growing evidence suggests that Diquat-induced intestinal injury is closely associated with dysfunction in distal organs through multiple molecular pathways. On one hand, microbial dysbiosis leads to reduced levels of key metabolites such as tryptophan and uridine, thereby compromising antioxidant defenses in peripheral organs (Christen et al., 1990). On the other hand, mitochondrial damage in intestinal epithelial cells can result in the release of mtDNA, which activates the cGAS–STING signaling pathway and triggers a systemic inflammatory storm (Newman and Shadel, 2023; Cai et al., 2024). Additionally, Diquat may interfere with autophagic flux—indicated by the abnormal accumulation of p62—facilitating the transport of misfolded proteins via exosomes and potentially inducing an unfolded protein response (UPR) in distal organs, although this hypothesis remains to be experimentally validated (Chen et al., 2021).

Given the central role of the intestine in Diquat toxicity, targeted intervention strategies show considerable potential. For example, resveratrol can enhance antioxidant capacity by activating the Nrf2 pathway and regulate AhR signaling to restore microbial metabolite homeostasis (Prakash et al., 2024; Meng et al., 2020). However, existing research has several limitations: first, most studies use acute high-dose exposure models, which fail to simulate the real-world scenario of low-dose chronic exposure in the environment; second, the spatiotemporal dynamics of microbiota-host metabolic interactions remain unclear; third, the strain-specific mechanisms of probiotics lack systematic comparative studies. Therefore, future research should incorporate new technologies such as organoid co-cultures and multi-omics analysis to elucidate the precise roles of key strains and metabolites (e.g., butyrate) in the detoxification process of Diquat.

The main strength of this study lies in its novel approach, systematically integrating the mechanisms of Diquat-induced intestinal toxicity from three dimensions: structural damage, microbial imbalance, and immune disruption. It introduces new hypotheses, such as “gut-derived ROS diffusion” and “microbiota-metabolite axis imbalance”, offering fresh perspectives that challenge the traditional liver and kidney-centered toxicological framework. This not only deepens our understanding of the inherent toxicity of bipyridyl herbicides but also provides a theoretical foundation for toxicity assessment and detoxification strategies.

Nevertheless, it is important to acknowledge the limitations of existing research: for example, human-related data is lacking, and most evidence comes from rodent models; the experimental models are relatively simplistic and fail to represent real-world scenarios of long-term low-dose exposure; the complexity of the gut microbiome is high, influenced by confounding factors such as diet and genetics, which complicates the interpretation of Diquat-specific effects; additionally, the selective mechanisms by which Diquat affects different microbiota remain unclear and may be related to differences in its structure’s ability to scavenge ROS or its sensitivity to metabolic pathways.

Future research should focus on the following key areas: first, the mechanisms by which Diquat selectively suppresses specific microbiota should be elucidated, exploring whether it causes dysbiosis through ROS tolerance, metabolic dependence, or membrane protein affinity; second, more realistic long-term low-dose exposure models should be developed to clarify the compensatory mechanisms of the intestinal barrier and the critical points of dysregulation; third, epidemiological studies on populations exposed to Diquat should be conducted to identify potential biomarkers, such as serum zonulin and microbiota profiles; finally, there is a need to develop responsive nanoparticle delivery systems to facilitate the targeted supplementation of key detoxification metabolites (e.g., indole-3-methanol), enhancing the precision and translational potential of intervention strategies.

In conclusion, the systemic toxicity induced by Diquat is essentially a reflection of the gradual progression of localized intestinal injury into a systemic pathological process. Adopting the “intestinal barrier-microbiota-immune” framework for research not only helps reinterpret its toxicological mechanisms but also provides a theoretical foundation for developing targeted detoxification strategies centered on the intestine. Even though Diquat has been banned in many regions, the “gut-organ axis” mechanism revealed by this “model toxin” still holds significant theoretical and practical value for environmental pollution management and ecological toxicology research.

## Abbreviations

AKI, acute kidney injury; EVs, extracellular vesicles; GSDME, gasdermin E; GSH-Px, glutathione peroxidase; MAM, mitochondria-associated membranes; MDA, malondialdehyde; MMP, mitochondrial membrane potential; MODS, multiple organ dysfunction syndrome; ROS, reactive oxygen species; SOD, superoxide dismutase.
